# DNA metabarcoding and its potential in microbial risk assessment in waste sorting plants

**DOI:** 10.1038/s41598-025-93697-9

**Published:** 2025-03-15

**Authors:** Elke Eriksen, Pål Graff, Alexander Eiler, Anne Straumfors, Anani Komlavi Afanou

**Affiliations:** 1 STAMI, National Institute of Occupational Health, Gydas Vei 8, 0363 Oslo, Norway; 2 Section for Aquatic Biology and Toxicology, Department of Biosciences, Centre for Biogeochemistry in the Anthropocene, University of Oslo, 0316 Oslo, Norway

**Keywords:** Occupational exposure, High-throughput sequencing, Human pathogens, Bacteria, Fungi, High-throughput screening, Sequencing, Occupational health, Bacteria, Fungi, Pathogens

## Abstract

Exposure to hazardous microorganisms during waste handling is a potential health concern. Molecular biological techniques provide means of profiling the microbial community at high taxonomic resolution, allow the identification of critical human pathogens on the species level and thereby aid the risk assessment of work tasks. The present study used high-throughput sequencing to characterise the microbiome in personal full-shift air samples collected at contemporary waste sorting plants (WSPs) and identified large variations in community composition within (alpha diversity) and between (beta diversity) WSPs. Seasonality did not contribute to differences in the community composition. *Cladosporium sp*. was dominant among fungi, whereas *Aerococcus sp*. was dominant among bacteria. The personal air-samples contained potential human pathogens, such as *Aspergillus sp.*, *Fusarium sp.* and Enterobacteriaceae, that encompass strains with the potential to develop drug-resistance. This study provided characterization of the microbial community composition of personal bioaerosol samples and provided evidence for the occurrence of potential human pathogens in contemporary waste sorting plants. Furthermore, this study highlighted the potential of microbial metabarcoding to detect critical human pathogens that may be encountered in working environments.

## Introduction

Waste workers are regularly exposed to organic dust containing microorganisms, microbial fragments as well as associated metabolites that may contribute to the development of occupational diseases. Occupational exposure to high levels of microorganisms has previously been associated with a diverse spectrum of adverse health outcomes, such as respiratory disorders^1^ and allergic symptoms^2^. However, due to the complexity of bioaerosols and the high variability of the airborne microbiome, exposure-related health effects are difficult to study and often inconclusive.

Previous research of air-borne microorganisms in waste sorting has focused on cultivable organisms and identified *Aspergillus* spp., *Penicillium* spp. and *Cladosporium* spp^3^. to be among the most prevalent fungal genera, whereas bacterial genera were dominated by *Staphylococcus sp*. and *Bacillus* spp^4–6^. Resent research indicated that culture-based studies potentially underestimated the microbial biodiversity as only approximately 35% of microorganisms are cultivable under beneficial conditions^7^. Cyprowski and colleagues^8^ used endpoint PCR analysis with primers specific for *Clostridium* in combination with microbiological techniques to identify viable microorganisms and found high bacterial loads in a Polish waste sorting plant. In a recent study Viegas and colleagues^9^ used comparable study approaches to characterise the fungal burden in work environmental samples from waste sorting plants and identified of *Aspergillus sp.*, the main cause of aspergilloses in humans, in all samples. However, as not all species within a genus pose a health risk, and endpoint PCR does not allow identification on the species level, the extent of non-viable human pathogens in these studies remains unclear.

High-throughput sequencing (HTS) facilitates the assessment of the composition of microbial communities at high taxonomic resolution, quickly, efficiently, and specifically. HTS-based profiling of microorganisms is readily applied environmental studies to identify invasive species^10^ as well as in clinical settings in order to monitor biological changes in the microbiome in association with disease^11^ or to assess the risk associated with potential human pathogens^12^. HTS allows the identification of microorganisms with immune-stimulatory potential on the species level and amplification of specific gene regions, such as the fungal internal transcribed spacer (ITS) region and the bacterial 6 S rRNA has enhanced the profiling of the microbial community in occupational settings and helped establish associations between fungal exposure and respiratory disease^13^. However, so far only a few studies have characterised the microbial biodiversity in waste sorting working environments using HTS approaches and the association between the composition of the microbiome and potential exposure related health effects remains largely unknown. Szulc and colleagues^14^ assessed microbiological hazards in Polish waste sorting plants and identified approximately 200 bacterial and fungal genera in work air samples. Furthermore, Degois and colleagues^15^ studied the microbial biodiversity in waste sorting plants and reported substantially higher bacterial diversity based on DNA profiling using HTS compared to previous studies that used culture-based methods. These studies highlight the need for HTS-based monitoring of work-exposure scenarios in order to identify potentially hazardous microorganisms on the species level.

The present study generated knowledge on the composition of the microbial community in contemporary waste sorting plants by using HTS that provides improved profiling of the air-borne microbiome. Studying the composition of the inhalable microbiome aids a better understanding of risks, such as the presence of potentially human pathogens, that may be encountered in waste handling and consequently helps maintain workers’ health.

## Results

### Amplicon sequences and taxonomy

The final dataset including quality filtered amplicon sequences of forward and reverse reads contained 63 637 merged non-chimeric bacterial reads (20 881 unique amplicon sequence variants (ASV), 2310 unique ASVs on the species level), and 30 345 merged non-chimeric fungal reads (2 231 unique ASVs, 1796 unique ASVs on the species level) in 112 personal air samples. Only ASVs that were identified at the species level were included in downstream analysis. 86% of the merged reads had an amplicon length above 230 bp. The mean number of ASVs per sample was 327 (min: 91, max: 571) for fungi and 568 (min: 47, max: 1389) for bacteria.

Abundance of microbial ASVs.

**Fig. 1:**
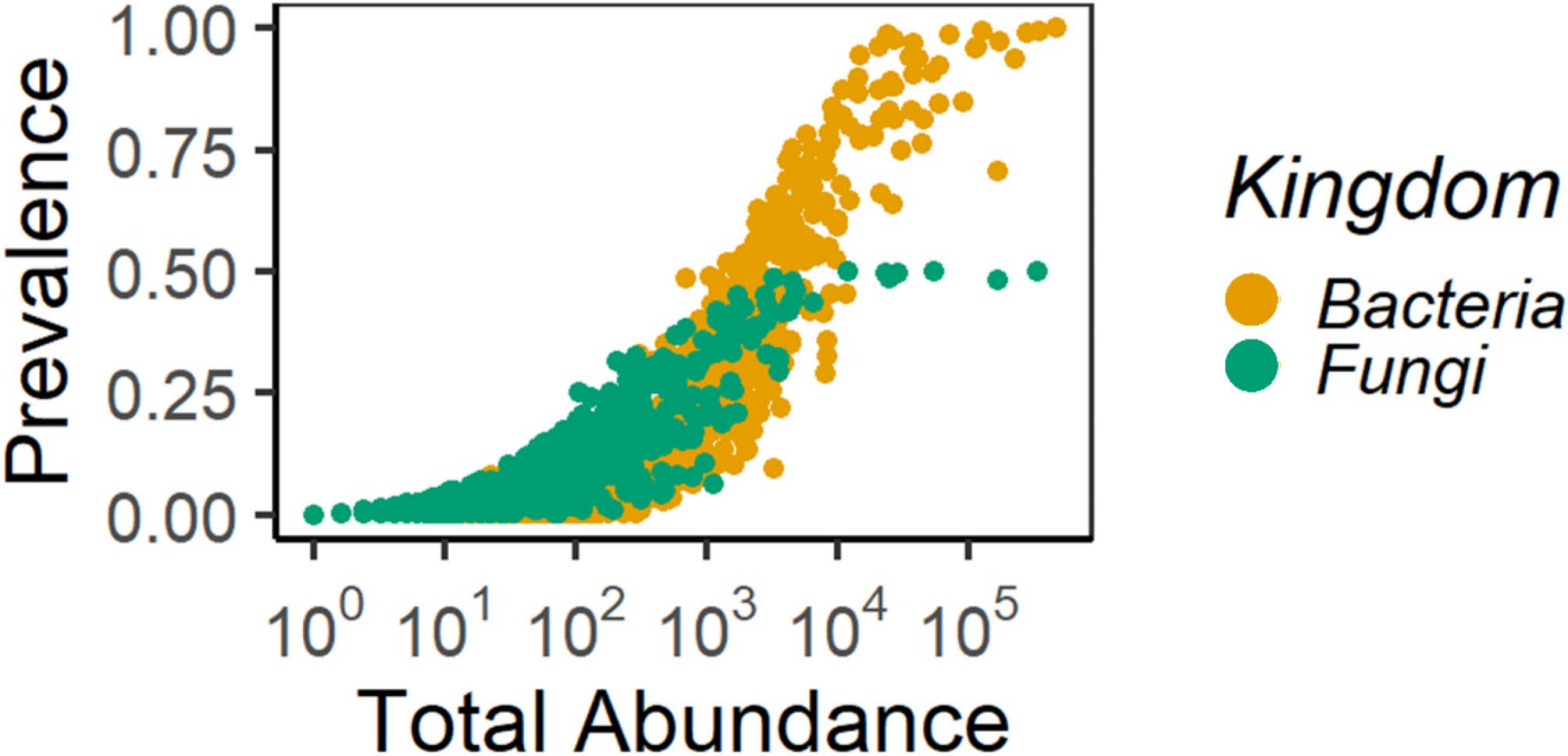
Prevalence / abundance plot of all identified ASVs in personal air-samples. Bacteria: orange, fungi: green.

Across all identified ASVs in the personal air samples, bacteria accounted for 68% of the microbial load, whereas fungi accounted for approximately 32% (Figure S1). A few ASVs of Archaea (0.16%) and other Eukarya (0.11%) were identified, these were however removed from the dataset prior to downstream analyses. Bacterial and fungal communities were characterised by few highly abundant ASVs and many rare ASVs (Fig. 1). 8 fungal ASVs, belonging to 4 genera (*Cladosporium*, *Candida*, *Clavispora*, *Penicillium*) were common in all samples, whereas only 2 bacterial ASVs (*Aerococcus urinaeequi*, *Lactococcus lactis*) were shared among all samples.

### Alpha diversity

**Fig. 2:**
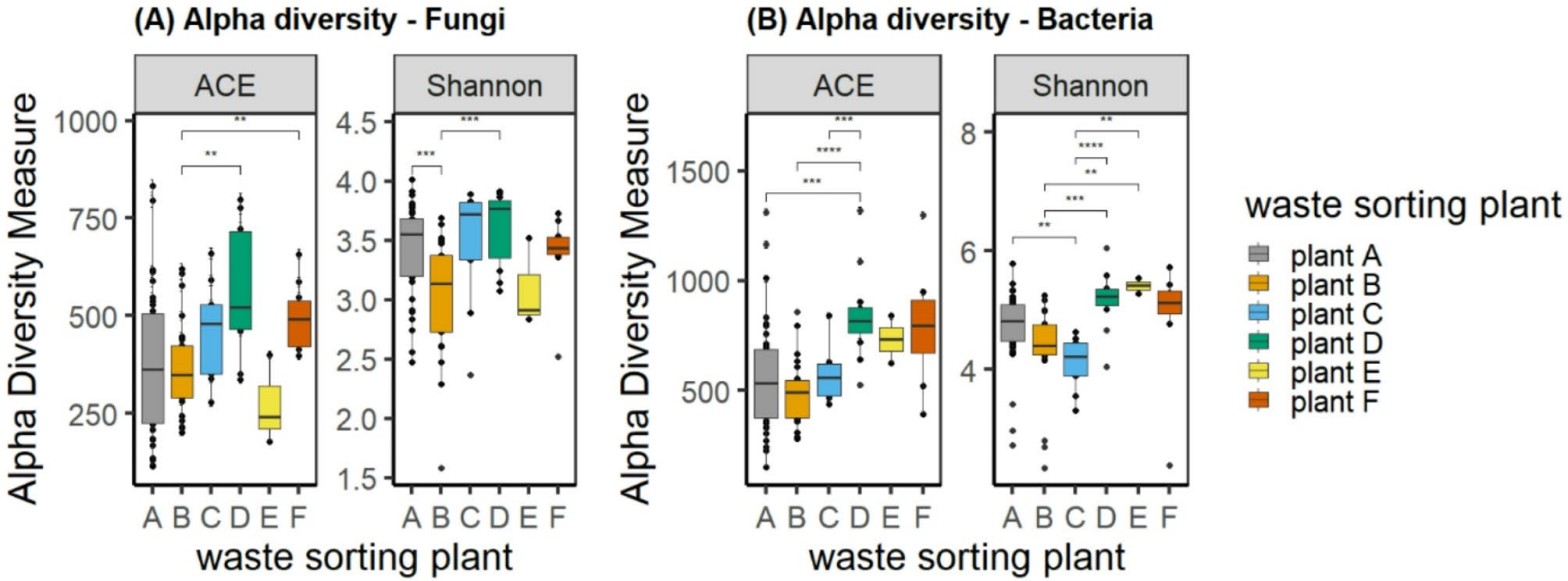
Alpha diversity in fungal (**A**) and bacterial (**B**) communities in personal air samples collected during autumn stratified by waste sorting plant. ACE shows community richness, Shannon indicates community diversity. A t-test was used to investigate the statistical difference in alpha diversity between wsp. Significance levels: * <0.05, ** <0.01, *** <0.001, ****<0.0001.

**Fig. 3:**
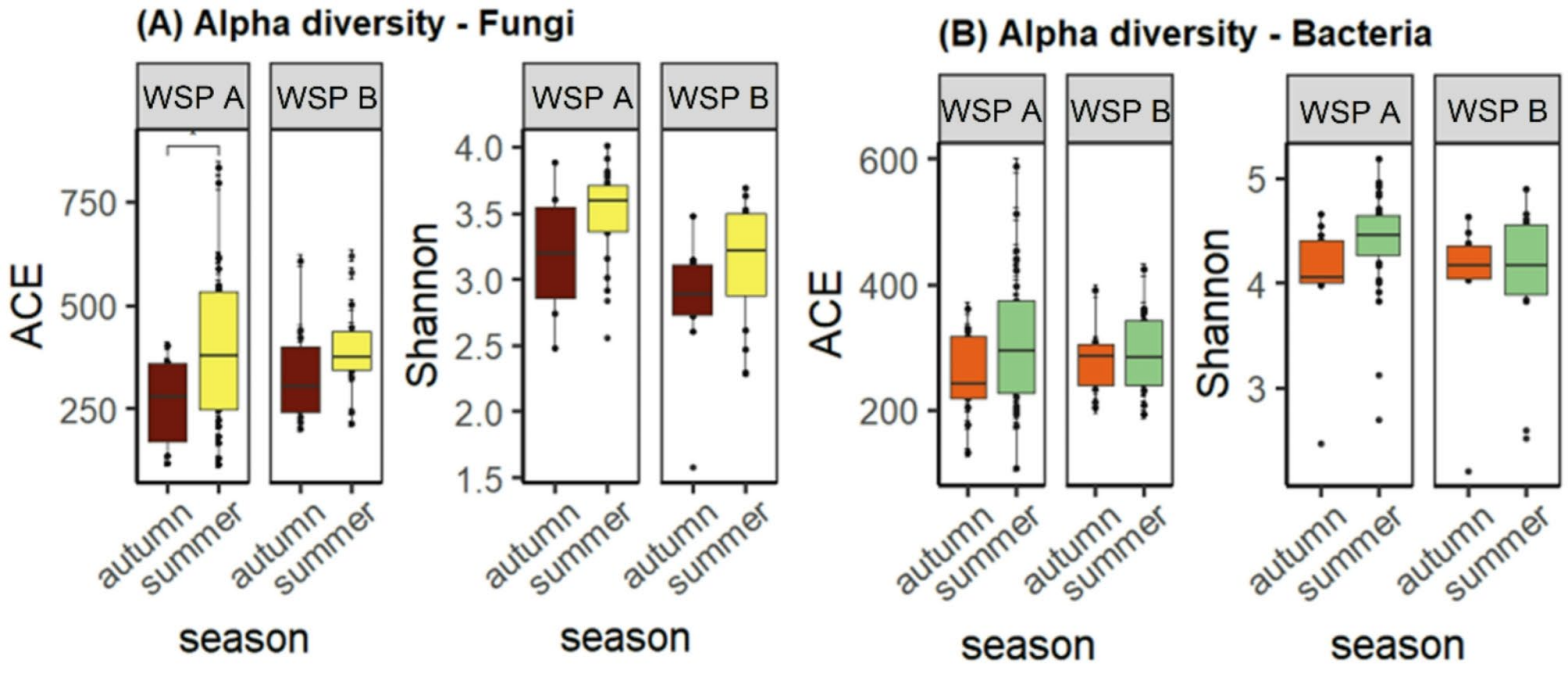
Seasonal differences in species richness and diversity for fungal (**A**) and bacterial (**B**) communities in personal air samples. ACE shows community richness, Shannon indicates community diversity. A t-test was used to investigate the statistical difference in alpha diversity between seasons. Significance levels: * <0.05.

The rarefied dataset included 1.1 × 10^7^ fungal and 1.6 × 10^7^ bacterial reads. Fungal species richness, measured as Abundance-based coverage estimator (ACE) differed significantly between WSP B and D and F, respectively. Highest richness was observed at plant D and lowest at plant E (Fig. 2A). The ACE in bacterial communities (Fig. 2B) was highest at plant D and differed significantly between plant D and plant A, B and C, respectively. The Shannon index indicates relatively high species diversity at the individual WSP. Diversity was generally higher in bacterial communities compared to fungal communities. Microbial communities were generally characterised by higher richness and diversity in samples collected during summer compared to autumn samples in plant A and B (Fig. 3A and B), the differences were however mainly not statistically significant. Significant seasonal differences in fungal communities were identified at WSP A, with higher richness (ACE) in summer samples (Fig. 3A).

### Beta diversity

**Fig. 4:**
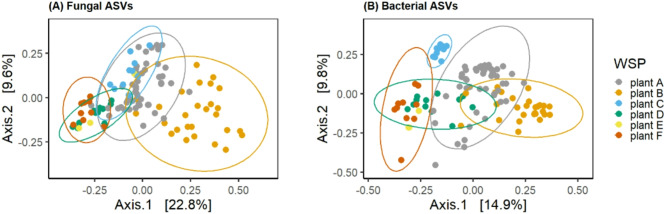
Beta diversity in personal samples at plant A (grey), plant B (orange), plant C (aqua), plant D (green), plant E (yellow and plant F (red). Stress score fungal ASVs = 0.24 bacterial ASVs = 0.17.

Ordination of fungal (Fig. 4A) and bacterial (Fig. 4B) communities in personal air samples. Permutation analysis of fungal communities (1000 permutations) revealed significant differences in beta diversity between WSP A and F, WSP B and C and WSP B and F (p value < 0.05). Bacterial communities did not differ significantly between WSP.

The fungal community was dominated by two phyla: Ascomycota (90%) and Basidiomycota (10%) (Figure S2A). Whereas a total of 49 different bacterial phyla was identified (top 15 showed in Figure S2B). *Firmicutes* were the dominant phylum at all WSP accounting for more than 40% of bacterial phyla, whereas bacterial ASVs at plant E, were dominated by *Proteobacteria* and *Bacteriodota* (35%. respectively).

**Fig. 5:**
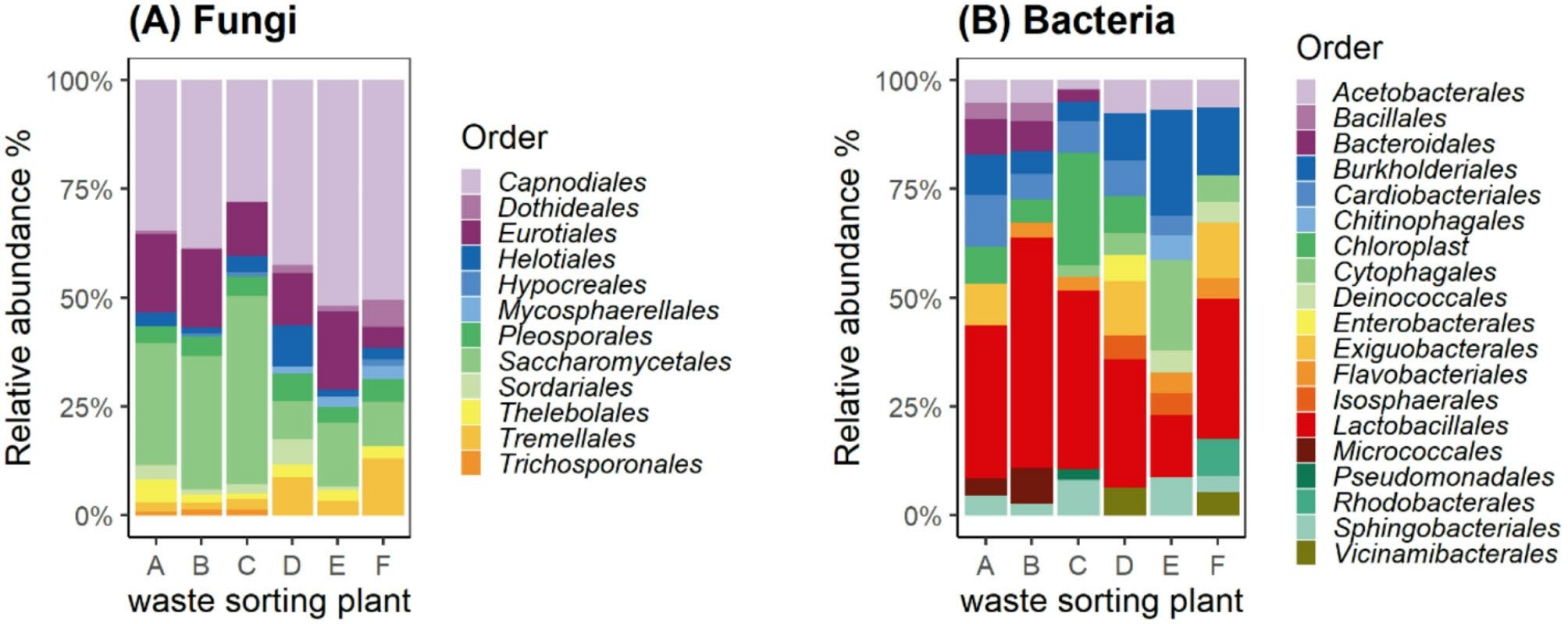
Abundance of the top 10 fungal (**A**) and bacterial (**B**) ASVs per waste sorting plant on the order level.

On the order level fungal communities were dominated by *Capnoidales* and *Saccharomycetales* accounting for over 50% of the identified ASVs at all WSP (Fig. 5A). The bacterial communities were dominated by *Lactobacillales* at all WSP except plant E., where *Burkholderidales* were the most abundant order (Fig. 5B).

**Fig. 6:**
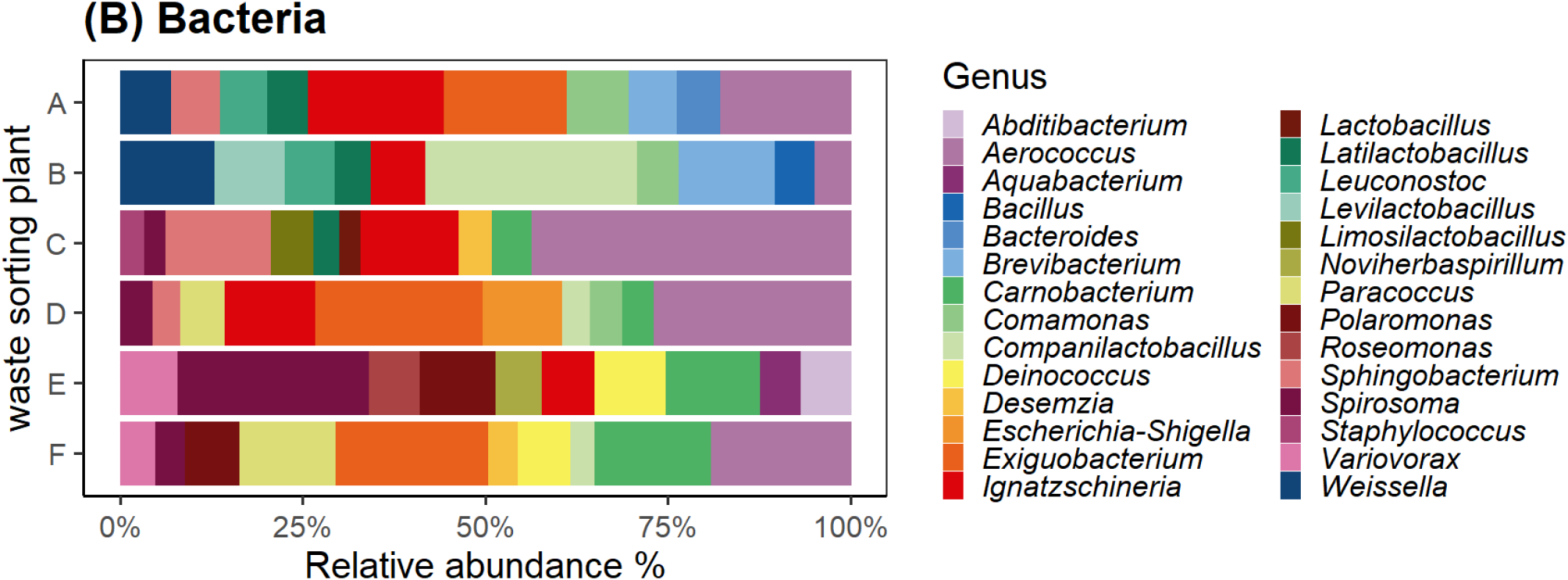
(**A**) fungal and (**B**) bacterial communities on the Genus level. Top 10 genera per WSP shown in the graph.

A total of 695 different fungal and 1145 different bacterial genera were identified in the samples. The 10 most abundant bacterial genera accounted for 53% of the total bacterial diversity, whereas the 10 most abundant fungal genera accounted for 90% of the total fungal biodiversity (Fig. 6). Among fungi, *Cladosporium* (37%), *Candida* (12%) and *Penicillium* (11%) were the most abundant genera, whereas *Aerococcus* (7%), *Ignatzschineria* (5%) and *Companilactobacillus* (5%) were the most abundant bacterial genera. *Candida*, *Cladosporium*, *Clavispora*, *Penicillium* and *Vishniacozyma* were identified in all WSP. None of the most abundant bacterial genera were shared among all WSP (Figure S3B).

**Fig. 7:**
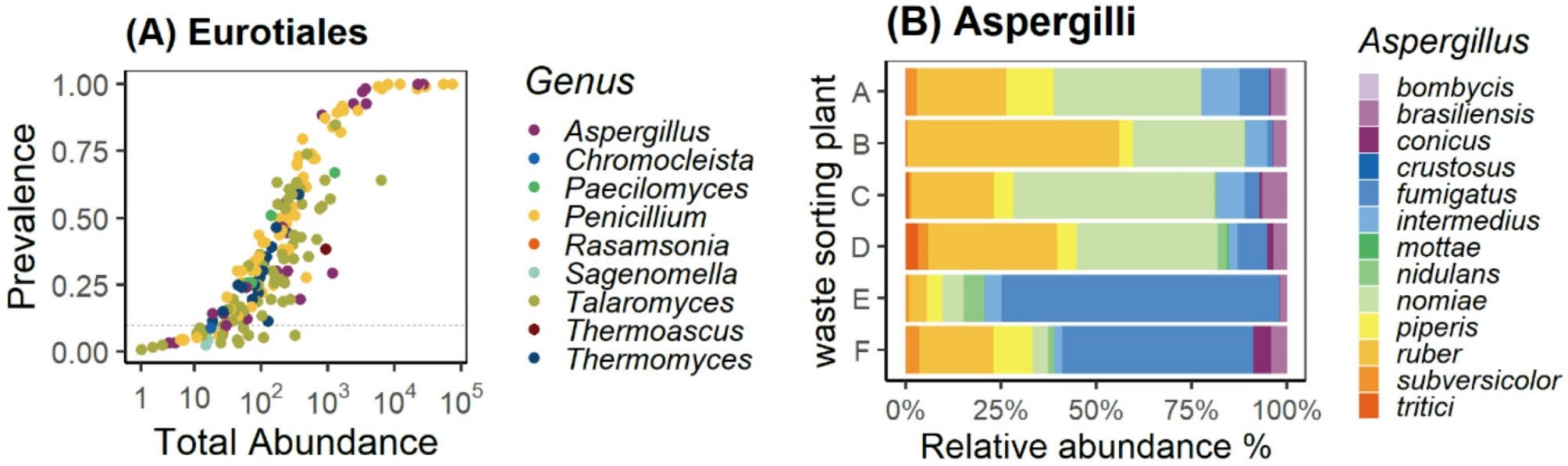
Prevalence/abundance of potential human pathogens such as Eurotiales (**A**) and species in the genus Aspergillus (**B**) in personal air samples.

The taxonomic order *Eurotiales* includes Genera such as *Aspergillus*,* Penicillium* and *Talaromyces*, which were the three most abundant and prevalent genera, *Chromocleista* and *Rasamsonia* were less prevalent (Fig. 7A, Figure S4). A total of 21 unique ASVs in the genus *Aspergillus were present in the dataset;* thirteen of these (62%) were identified on the species level and accounted for 2.5% of all fungal ASVs in personal air samples (Fig. 7B). At WSP A to D, the species *A. ruber* and *A. nomiae* dominated among *Aspergilli*, whereas *A. fumigatus* dominated the fungal ASVs in WSP E and F in autumn samples. Furthermore, the samples contained 11 different species belonging to the genus *Cladosporium* (Figure S7) which corresponds to 17% of all unique ASVs in the genus that were identified (65 unique ASV belonging to genus *Cladosporium).*

**Fig. 8:**
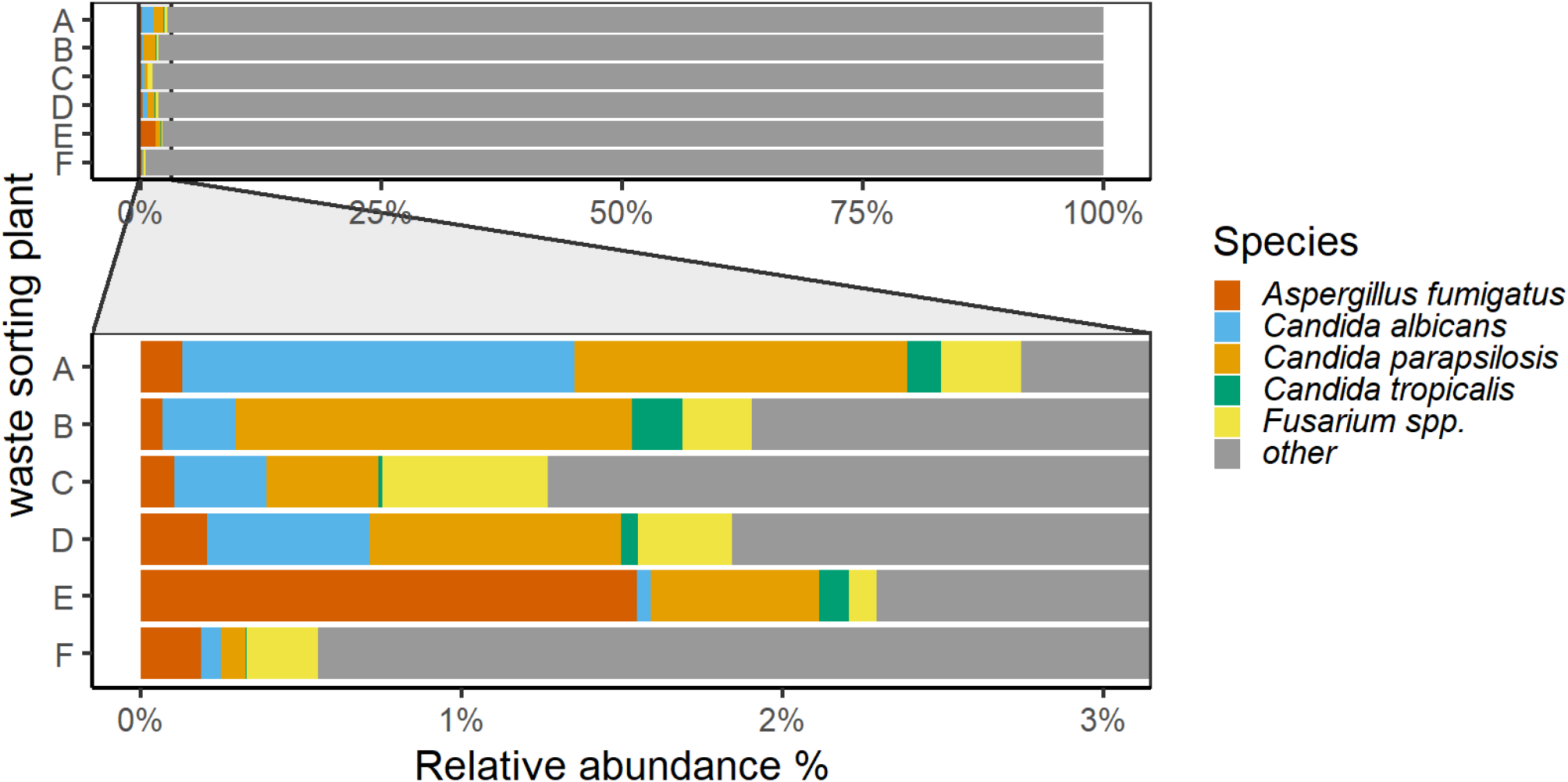
Abundance of WHO classified critical human pathogens in the personal bioaerosol samples collected at six waste sorting plants (A: F).

Five of 19 fungi that are included in WHO’s priority pathogen list (Fig. 8) were identified in personal air samples and accounted in total for approximately 2% of all identified fungal ASVs. *Aspergillus fumigatus*, *Candida albicans*, *C. parapsilosis*, *C. tropicalis* as well as 28 unique ASVs on of the genus *Fusarium* were identified. Sixteen of these were identified on the species level.(Figure S5). The most abundant species were *Fusarium tricinctum* (28%), *F. anthophilum* (16%) and *F. oxysporum* (15%) that accounted for over 50% of the identified species of the genus *Fusarium*.

### Potentially multi-drug resistant bacteria

**Fig. 9:**
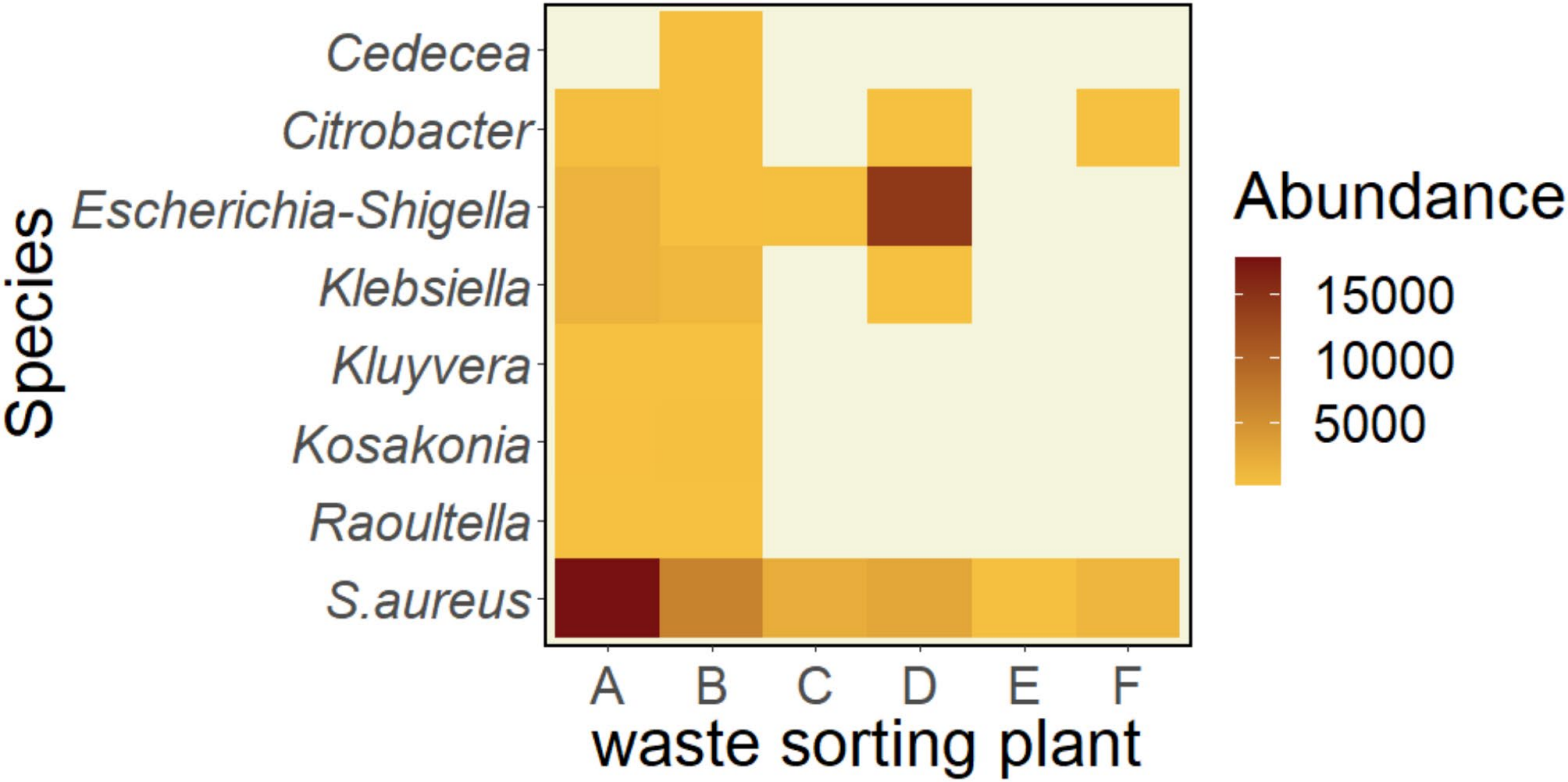
Heatmap of potentially multi-drug resistant bacteria.

Seven different genera belonging to the family *Enterobacteriaceae* (*Cedecea*,* Citerobacter*,* Escherichia-Shigella*,* Klebsiella*,* Kluyvera*,* Kosakonia* and *Raoultella*) were identified accounting for 0.6% of the total bacterial ASVs (Fig. 9). One per cent of the identified bacterial ASVs were assigned to *Staphylococcus aureus*. *S.aureus* was identified at all WSP with highest abundance at plant A.

## Discussion

The present study used HTS approaches for microbial identification and utilised primer sets that covered a wide range of bacteria and fungi, respectively and thereby provided insights in the occupational microbiome at six contemporary waste sorting plants. The microbiome contained 1.7 times higher number of bacterial ASVs compared to fungi, the microbial community composition, however, varied greatly between waste sorting plants. Seasonality did not affect the microbial communities. Personal bioaerosol samples contained potential human pathogens and multi-drug resistant microorganisms.

Potential of HTS based community profiling.

Culture-based methods remain a cornerstone for the identification of culturable microorganisms as they allow the detailed study of microbial physiology, antibiotic resistance, and metabolic capabilities. However, these methods are limited by their inability to detect non-culturable organisms or fragments thereof. HTS-based monitoring allows the detection of a broader range of microbial species, including non-culturable and low abundant organisms, and thereby provides a more comprehensive view of the microbial community^16^.

HTS approaches are readily used in environmental DNA profiling and clinical research to identify potentially problematic organisms^10–12^. In occupational exposure studies, however, modern molecular techniques are still little used. Previous research on microbial exposures in the waste industry has used PCR approaches to investigate the microbial biodiversity in work-environmental air-samples and provided valuable insights in community composition on superordinate taxonomic levels^8,17^. Such investigations may allow the identification of potential human pathogens, such as species in the genus *Aspergillus* that may be of relevance as indicator species for pathogenic fungal exposure as proposed by Viegas and colleagues^18,19^. The present study identified 2310 unique bacterial and 1796 unique fungal ASV on the species level, which corresponds to 11% and 80% of the total bacterial and fungal ASV in the dataset. This indicates that a substantial fraction of the microbiome, especially for bacteria, could not be investigated at high taxonomic resolution which complicates the study of the total potential of work-environmental air-samples. This limitation may be due to the sequencing effort, such as the choice of primers. Closely related species often express high sequence similarity, particularly in conserved regions such as the 16 S rRNA, which poses significant challenges to identifying ASVs on the species level^20^. Furthermore, the presented dataset used single sequencing depth which may be insufficient to capture the full diversity of the microbiome. Higher sequencing depth is preferred to capture less abundant species and enhance accuracy by minimising sequencing errors^21,22^, which should be considered in future studies.

As pathogenicity is often highly variable within a genus and associated with single species or strains, it is relevant to investigate the microbiome at high taxonomic resolution in order to identify human pathogens such as *Aspergillus fumigatus* (Fig. 7), the main cause of aspergillosis in humans^23^. Other fungi, such as species in the genus *Fusarium* (Fig. 8, Figure S5), a filamentous fungus and common plant pathogen have been described to be of clinical relevance in their role as opportunistic human pathogens^24^. Due to their partial resistance to azoles, a group of therapeutic antimycotics, as well as their mycotoxin producing abilities infections with specific species or strains may be a major health concern especially in immune-compromised individuals^25,26^ in which aggressive fusariosis caused by species in the *F. solani* complex may cause systemic infections and infections of the blood stream^25^. As potential health-impairing microorganisms, such as *Fusarium* spp. are omnipresent in the environment and may be causative agents for occupational disease, the utilisation of HTS profiling of bioaerosols in work environmental settings is highly relevant in order to risk assess workplaces and maintain workers’ health.

### Biodiversity and community composition

The present study showed high variability in alpha diversity measures, both richness and evenness, within and between WSP, indicating that the microbiome is highly variable between work tasks within a plant as well as between workplaces (Fig. 2). High species richness was generally associated with work tasks that generated dust, such as cleaning operations and processing paper and cardboard which are work tasks that previously have been associated with high personal exposure^3,27^. Species richness and evenness tended to be higher during summer, but the differences were statistically not significant, except for fungi at WSP A, which showed higher levels of species richness during summer months (Fig. 3). The lack of a seasonal effect on richness and diversity suggests that the composition of the bacterial communities was affected by differences in the incoming waste material and executed work operations, rather than seasonality. Environmental factors were not measured in this study, and temperature data from nearby meteorological sites during sampling were used (Table S2). The data showed minor seasonal temperature differences: 8–12 °C at plant A and 11–15 °C at plant B, supporting the assumption that the observed variation was due to waste material rather than environmental factors. Seasonality has previously been shown to be a contributing factor to increased microbial exposure during warmer seasons at waste sorting plants^3,28^. These studies, however, assessed viable bacteria and fungi, as well as endotoxins and may thus be not directly comparable to exposure measurements obtained by sequencing approaches, as only a fraction of microorganisms are cultivable^29^ or endotoxin-producing^30^.

There is only a limited number of studies that have assessed bacterial and fungal biodiversity using HTS approaches in the waste industry. Recent research has used study approaches comparable to the present study to investigate the microbiome in the waste industry^14,31,32^. Szulc^14^ reported high proportions of *Actinobacteria*, *Firmicutes* and *Acidobacteria* in air samples collected at Polish waste sorting plants, whereas the present study identified the majority of bacteria to belong to the phyla *Firmicutes*, *Proteobacteria*, and *Bacteriota* (Figure S2). Duquenne and colleagues^31^ studied the microbiome in air samples collected at municipal solid waste plants and reported that the fungal community comprised of on average 85% Ascomycota and 5% Basidomycota. The top 15 most abundant fungal genera accounted for 92% of the total fungal community, and *Penicillium* and *Cladosporium* were the most prevalent genera. Similar observations were made in the present study, in which the top 20 fungal genera accounted for 90% of the total fungal biodiversity (Fig. 6), and *Cladosporium sp.* were dominant at all WSP accounting for 37% of the fungi, indicating, that the microbiome at different workplaces is dominated by few highly abundant species. In a study published by Mbareche and colleagues^32^ *Cladosporium* was among the least abundant in samples collected at composting plants. In this study, airborne fungi belonged predominantly to the class *Eurotiomycetes* and *Saccaromycetes*. These divergent results concerning dominant genera may be partly due to the choice of sampling equipment, the choice of primers, but also environmental variations. Whereas Duquenne and the present study used filter-based sampling, Mbareche sampled in a liquid medium. It can be assumed, that the differences in the sampling efficiency between impaction and impingement promoted the collection and conservation of different airborne fungi and thus affected the recovered microbiome^33^.The present study revealed substantial variation in species composition between the assessed waste sorting plants, indicating that the microbiome appears plant specific, rather than generally representative for the waste sorting industry. Thus, the microbiome presented in other studies that assessed individual plants, may not be comparable to the present study that included six very diverse waste sorting plants and thereby reflects the conditions met in contemporary waste work environments in a different way.

### The role of human pathogens and multi-drug resistant microorganisms

The identification of human pathogens and microorganisms with health impairing potential is crucial in work environmental settings in order to prevent illness and promote workers’ health. The present study showed that personal air samples contained diverse spectra of human pathogens and potential toxin-producing microorganisms, such as *Staphylococcus aureus* that was present in 80% of all personal air samples and accounted for 1% of the identified bacterial ASVs (Fig. 9). In addition to its role as toxin producer, *S. aureus* is listed on the WHO high priority list of multi-drug resistant organisms^34^ due to the species’ frequent resistance to methicillin and vancomycin and thereby may constitute a major health concern^35,36^. Additionally, Enterobacteriaceae were identified in 50% of all samples and accounted for 0.6% of the bacterial diversity (Figure S6). Enterobacteriaceae including species such as *E.coli* and *Klebsiella pneumoniae*, are a normal part of the gut-flora, however, these may display multi-drug resistance due to their ability to produce extended spectrum beta-lactamase (ESBL), an enzyme that is able to break down commonly used antibiotics^37^. The presence of Enterobacteriaceae in wastewater has previously been discussed as contributor to the development of multi-drug resistance as wastewater provides perfect conditions for horizontal gene-transfer of resistant genes^38–40^. Korzeniewska and Harnisz^40^ investigated the emission of ESBL-positive Enterobacteriaceae from sewage plants and recovered 33% of the ESBL-producers in river water and 23% in environmental air samples that were collected in close proximity to the plants, and thereby provided evidence that ESBL-producing bacteria were emitted from sewage to the environment. ESBL-producing bacteria may cause pulmonary infections, as well as sepsis and gastrointestinal and urinary tract infections^37,41^. Enterobacteriaceae and *S.aureus* are classified as critical and high priority bacteria in accordance with the WHO list of possible multi-drug resistant organisms and the presence of these in the work-environmental samples gives reason for awareness towards potential exposure related health effects. Furthermore, the WHO included 19 different fungi in the pathogen priority list^42^. Five of these were identified in the present study in all WSP, albeit only in few samples (Fig. 8, Figure S5). *Fusarium oxysporum*, an opportunistic pathogen that may infect immune-compromised persons, is of major health concern due to its partial resistance to azoles, a group of therapeutic antimycotics, and mycotoxin producing ability^24^. Toxin producing fungi and bacteria accounted for approximately 5% of all identified ASVs in the present study, such as *E. coli*,* K.pneumoniae*,* F.anthophilum*, *P.expansum* and *S.chararum* all of which have previously been associated with adverse health effects such as of the respiratory tract and skin infections^43^. Furthermore, 2.5% of the identified fungal ASVs belonged to the genus *Aspergillus* the main causative agent for aspergillosis, which is significantly different from results reported in other research that studied airborne fungi in waste sorting. Viegas^17^ demonstrated that *Aspergilli* were clearly the dominant genus in Portuguese waste sorting plants accounting for 99% of the airborne fungal biodiversity. Viegas and colleagues^17^ used specific primers to identify species in the *Aspergillus* section, thus, the results may not be directly comparable with the present study which targeted a wider spectrum of the fungal genome^44^ including a variety of clinically relevant fungi. The primers used in the present study limited the identified fungal microbiome to Ascomycota and Basidiomycota and excluded other fungal divisions such as Mucoromycota, some of which are included on the WHO priority list of fungal pathogens^45^. It remains unclear to what extent fungi from other divisions contributed to the total fungal microbiome in Norwegian waste sorting plants and should be included in future studies. Furthermore, it needs to be considered that the HTS approach facilitated the identification of inanimate microorganism whose pathogenicity may be limited. However, as previously reported, exposure to bioaerosols can vary substantially between work operations, and increased exposure has been investigated in association with cleaning with compressed air and manual sorting of paper and cardboard^3,28^. It is during such peak exposure operations that exposure reducing measures, such as increased ventilation or the use of respiratory protective equipment, should be taken in order to reduce personal exposure and prevent occupational disease. The present study identified human pathogens, such as *Aspergillus spp.*, in the work environment however, mainly at low prevalence and in individual samples only. This emphasizes that the microbiome within workplaces may be highly variable, task-dependent and that the microbial composition in in individual samples may not be indicative for the general exposure at a workplace. Additionally, as the microbiome is affected by environmental factors, sorting technologies and incoming waste material, the results presented in the current study may not be representative for exposures at other waste sorting plants and the health burden posed by human pathogens may be dissimilar in different occupational settings.

The present study provided insights in the microbiome encountered in the work environment at contemporary waste sorting plants and highlighted benefits of HTS-monitoring approaches in order to risk assess occupational exposure and maintain workers’ health.

### Shortcomings of the study

Albeit the study was conducted at six contemporary waste sorting plants, the executed work tasks, waste soring processes as well as the incoming waste material differed substantially between plants which impedes the comparability of the results to a certain extent. Furthermore, the effect of seasonality on the microbiome was evaluated based on repeated measurements at two waste sorting plants only. It remains unclear to what extent seasonality affected work exposure to microorganisms at the remaining four plants. Temperature and humidity were not recorded during the sampling campaigns, thus the presented references from meteorological sites close to the plants can be used as a proxy only (Table S2). Additionally, the assessed microbiome focused on indoor samples only and lacks the comparison to the outdoor microbiome. This is a major shortcoming of the study and should be avoided in future research. The present study used primers specific for the fungal ITS2 region^44^ and the bacterial 16 S rRNA V4-V5 region^46,47^. As the ITS2 region covers only a minor fragment of the fungal genome there may be shortcomings due to primer coverage in such a way that some groups may preferentially be amplified or not be amplified at all. Furthermore, it remains unclear to what extent the pre-amplification step added to a selection bias and overrepresentation of certain phyla. Rarefaction curves suggested that the sequencing approach was sufficient to capture full diversity for bacteria, however not for fungi, suggesting that single sequencing depth could have been insufficient for profiling the fungal community. The deficient species concept for microorganisms is a major concern for the phylogenetic assignment. Due to high intra- and interspecific sequence similarity taxonomic assignment can be insufficient, especially if region-specific primers, such as in the present study, are used. Alternative sequencing approaches, such as whole genome sequencing may be the wiser choice to capture full microbial biodiversity, especially for organisms with high intra-specific sequence variability.

## Conclusion

The large variation in alpha diversity observed in the present study indicates that personal exposure is task dependent. Certain work operations represented particularly high risk of microbial exposure. Work exposure was highest among workers who participated in work operations that aerosolised dust, such as cleaning with compressed air and sorting of paper/cardboard. It may be beneficial to reduce the use of compressed air during cleaning from a health perspective. Microbial communities were characteristic for the individual WSP. Some of the personal air samples contained of human pathogens, toxin producing fungi and bacteria and organisms that are listed on the WHO’s priority list of emerging pathogens and potential drug- resistant microbes. The exposure of these microbes at work may be of concern, especially in immune-compromised persons. The work environment was more affected by the composition of the incoming waste material and how this was handled at the individual plants, rather than seasonality. It remains unclear to what extent temperature and humidity contribute to differences in the microbiome. Further research is needed to disentangle the effect of seasonality on personal exposure.

## Materials and methods

### Study population

Sampling was conducted at six fully operative Norwegian waste sorting plants that participated during June 2020 and November 2021. A total of 71 waste workers participated on two consecutive workdays during the sampling campaigns and helped recover 114 personal air samples. Plants A to C were modern, fully-automated waste sorting facilities that primarily handle residual waste from domestic households. In contrast, Plants D to F were traditional facilities that sort waste from small businesses and housing communities manually, with the assistance of excavators. Detailed descriptions of the sampling strategy and study population are published elsewhere^3^.

### Sample collection, filter extraction and pre-amplification

Personal full-shift air samples were collected on two consecutive workdays using membrane polycarbonate filters with a pore size of 1 μm (Frisenette, DK) that were contained in conical inhalable samplers (CIS, JS Holdings, Hertfordshire, UK) and operated at an airflow of 3.5 Lmin^− 1^. Additionally outdoor background samples (placed approximately 50–100 m off site) were collected midweek during each sampling campaign. Air flow was measured prior to and after sampling. The sampling equipment was mounted on the strops of backpacks and placed in the workers’ breathing zone.

Exposed filters were extracted following the procedures described by Straumfors^48^. Bacterial and fungal DNA were recovered in one extraction step from the same filter using cell lysis in combination with spin-column separation (DNeasy Mini plant kit, Quiagen GmbH, Hilden, Germany). DNA concentrations were measured using a Qbit 4 Fluorometer (Thermo Scientific, DE, USA). DNA concentrations in outdoor samples was generally out of range or very low. Consequently, these samples were not included in downstream analysis. Template DNA concentrations in on-site samples varied from 0.021 ng µL^− 1^ to 15.2 ng µL^− 1^. DNA recovered from personal air samples was diluted such as to adjust the input template DNA concentration to vary between 0.5 pg µL^− 1^ and 2.5 pg µL^− 1^ per reaction volume. Pre-amplification of fungal ITS2 and bacterial V4-V5 was executed on an Eppendorf Mastercycler X50s (Eppendorf SE, Hamburg, Germany) under the following conditions: Initial denaturation at 90 °C for 30 s, followed by 30 cycles of amplification (98 °C for 10 s, 60 °C for 30 s and 72 °C for 30 s) and a final elongation step at 72 °C for 5 min. Each reaction contained 5 µL 5x HF PCR buffer, 0.5 µL dNTPs (40mM), 5 µL of each the forward and reverse primer, 0.25 µL Phusion polymerase (2U/µL), 7.25 µL PCR-grade water and 2 µL template DNA. The primer sets 515FB/926R^46,47^ and ITS86(F)/ITS4(R)^44^ were used for bacterial and fungal amplification, respectively (Table S1). PCR reactions were run in duplicates (25 µL each) and pooled after amplification. Subsequently PCR products were purified using a PCR Purification kit (Norgen Biotek Corp., Thorold, Canada) following the manufacturer’s recommendations. The PCR products were sequenced on a MiSeq amplicon sequencing platform (IMR, Halifax, Canada).

### Bioinformatics

Sequencing data were analysed in R Studio (version 2022.12.0) using the DADA2 package^49^. Forward and reverse sequences were processed separately and merged in a later step. To increase the quality of the data, sequences containing unidentified bases were removed. Forward and reverse primers were identified and removed using DADA2’s remove primer function. The truncation length was limited to 230 bases in the forward and 180 bases in the reverse sequences, and to a minimum of 50 bases for bacterial and fungal sequences, respectively. Amplicon sequences were clustered based on a global clustering threshold and forward and reverse sequences were matched enforcing a minimum overlap of 12 base pairs (bp) for bacteria and 5 bp for fungi, allowing zero mismatches between sequences. Reads with a quality score above 30 were included in downstream analyses. Forward and reverse sequences were denoised independently and subsequently merged allowing a 12 bp overlap for bacterial and 5 bp overlap for fungal sequences. Chimeric sequences were removed. Taxonomic assignment was achieved using the SILVA138.1 database for bacteria^50,51^ and the UNITE database for fungi^52^, respectively. A mock community was included during all steps in the protocol as sanity check. The amplicon data was analysed using the phyloseq^53^, vegan^54^, and microbiome^55^ packages and visualised using ggplot2^56^.

### Diversity analyses

Alpha diversity was investigated based on a rarefied dataset allowing for optimal sample coverage (sample size fungi: 11000, sample size bacteria: 15000. Species richness was reported as Abundance-based coverage estimator that accounts for rare ASVs in the dataset. A t-test was used to estimate differences in alpha diversity between wsp and seasons. Community diversity was reported as Shannon diversity. Permutational Multivariate Analysis of Variance (Permanova) (1000 permutations) using a Bray Curtis distance matric was used to estimate beta diversity between WSP and seasons. Differences in community composition were considered statistically significant at a p value < 0.05.

The WHO fungal priority pathogens list^42^, WHO Bacterial Priority Pathogens List^34^ were used as reference tools for identification of potential human pathogens. The IFA GESTIS database^57^ was used as reference tool for the identification of toxin producing microorganisms.

## Electronic supplementary material

Below is the link to the electronic supplementary material.

Supplementary Material 1

## Data Availability

Data will be made available upon reasonable request. Please contact elke.eriksen@stami.no.
