# Supplementary material for: DNA metabarcoding and its potential in microbial risk assessment in waste sorting plants
Source: Sci Rep. 2025 Mar 15;15:8941. doi: 10.1038/s41598-025-93697-9 (PMC11910513; doi:10.1038/s41598-025-93697-9)
Supplement: Supplementary file 1 — Supplementary Material 1 [file 41598_2025_93697_MOESM1_ESM.docx]

Paper supplement

**DNA metabarcoding and its potential in microbial risk assessment in waste-sorting plants**


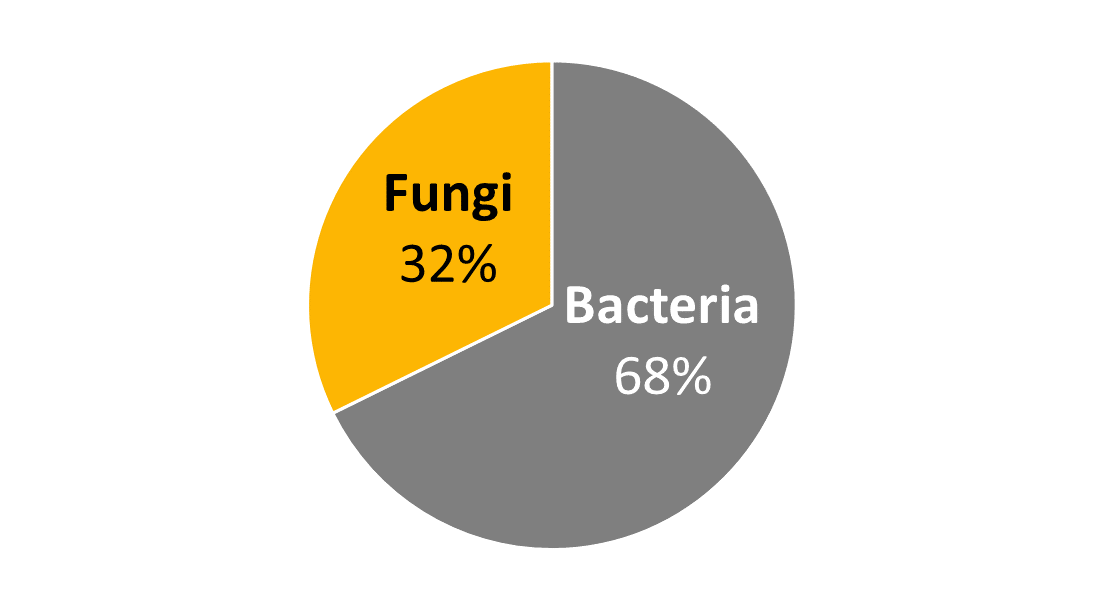

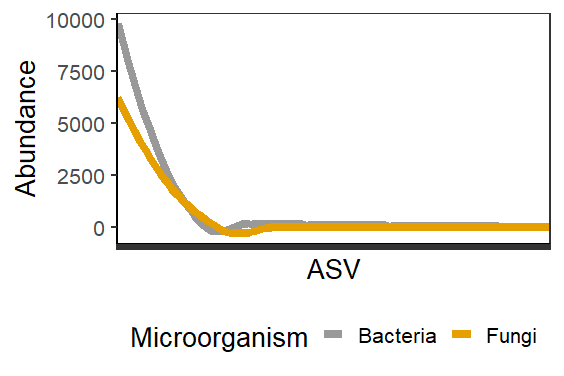

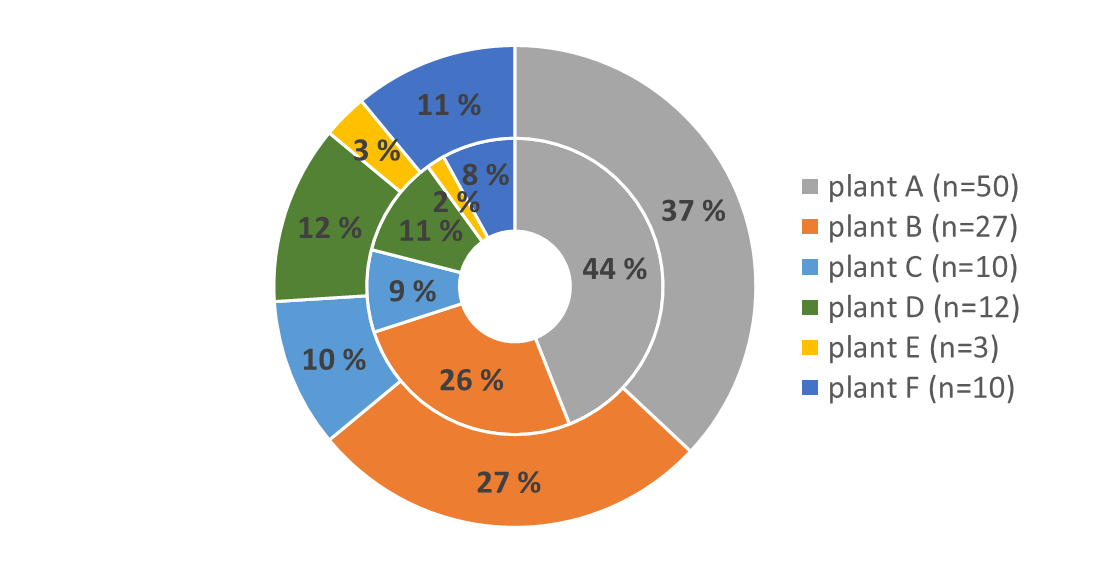


Figure S1 - Abundance curves for fungal (orange) and bacterial (grey) ASVs and proportion of total ASVs in personal air samples. Per cent of total identified fungal ASVs (outer circle) and bacterial ASVs (inner circle) by waste sorting plant.

### Microbial abundance


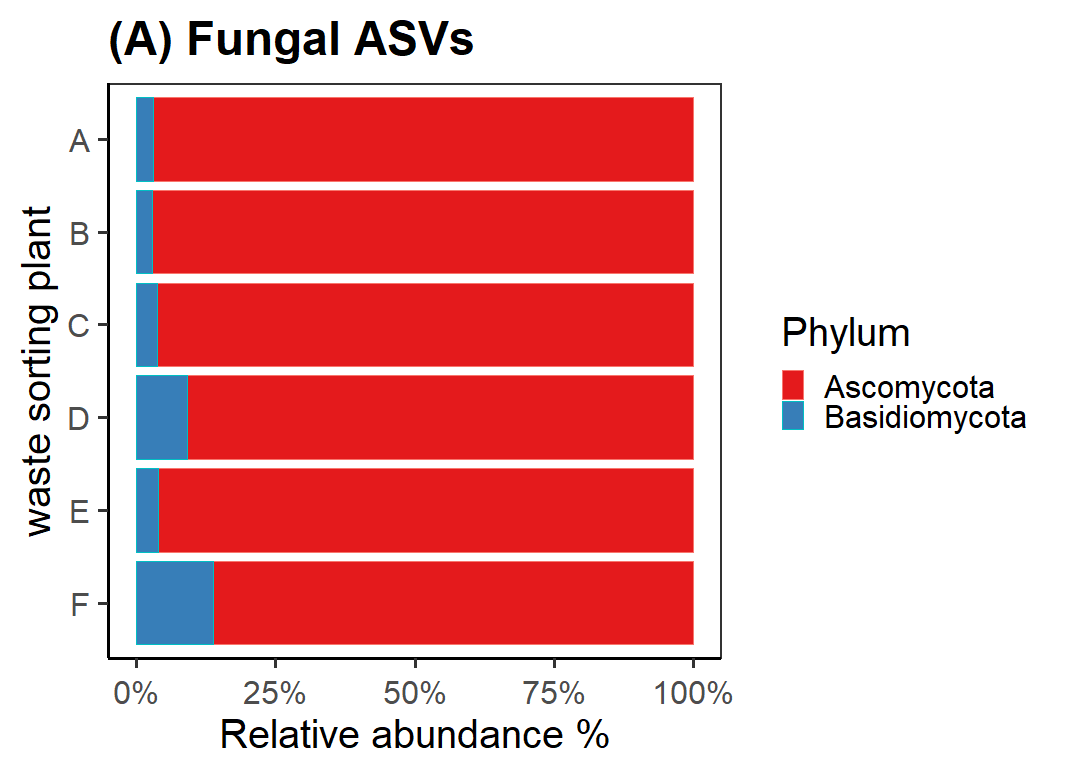

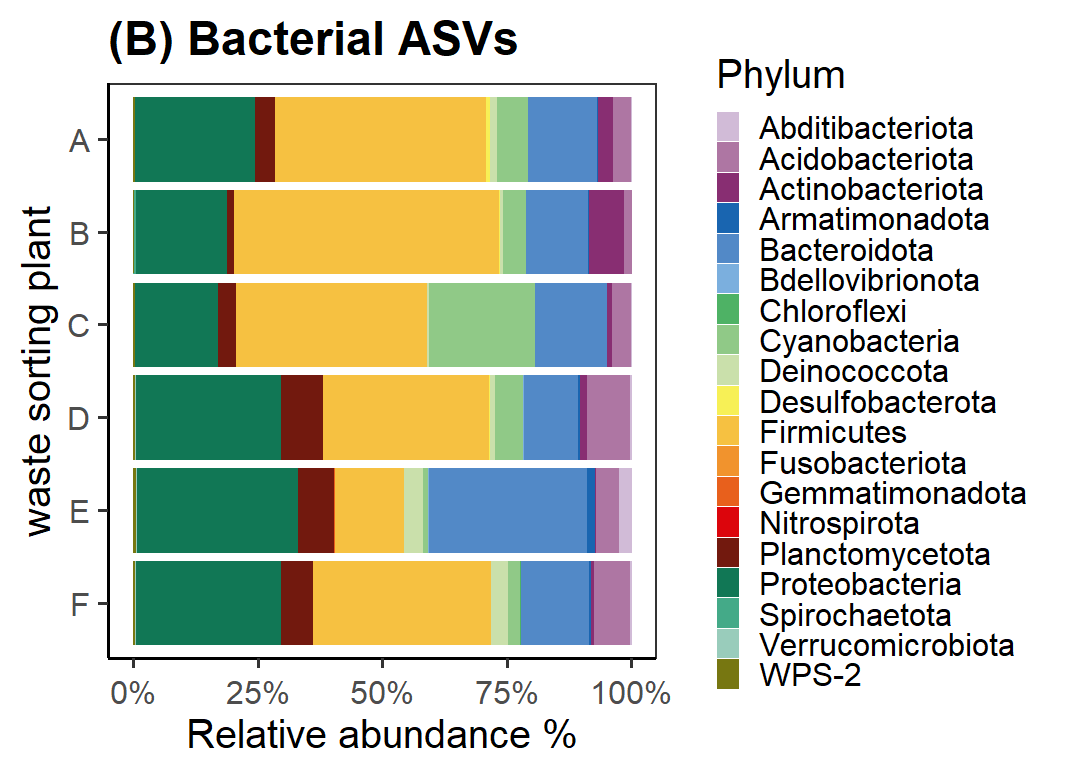


Figure S2 - Abundance of fungal (A) and top 15 bacterial (B) phyla in personal air samples.


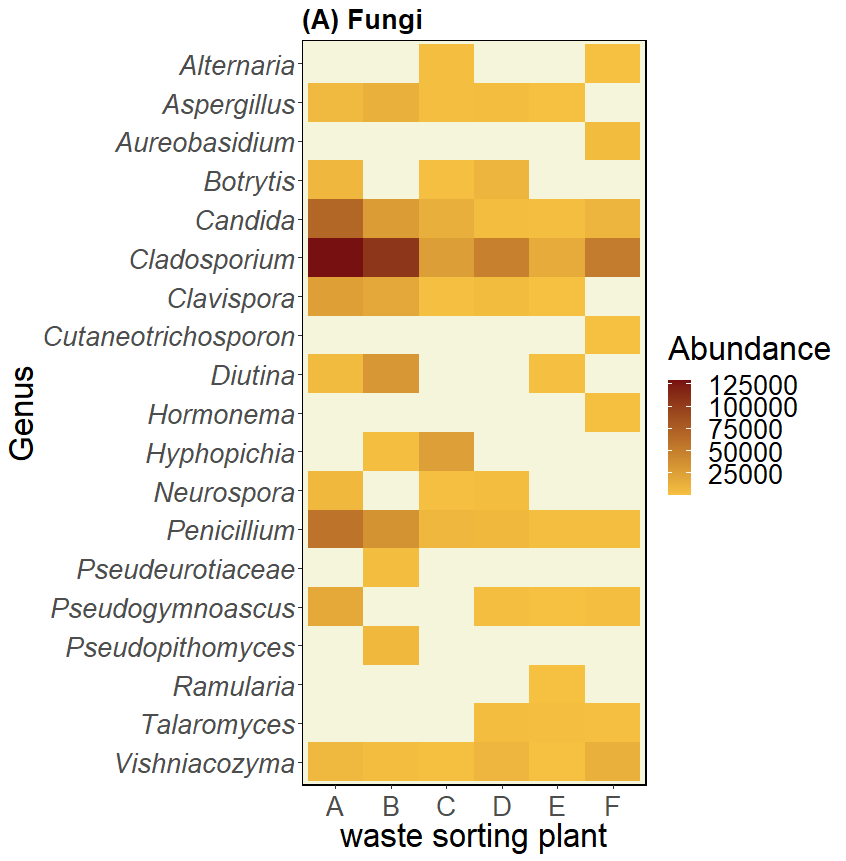

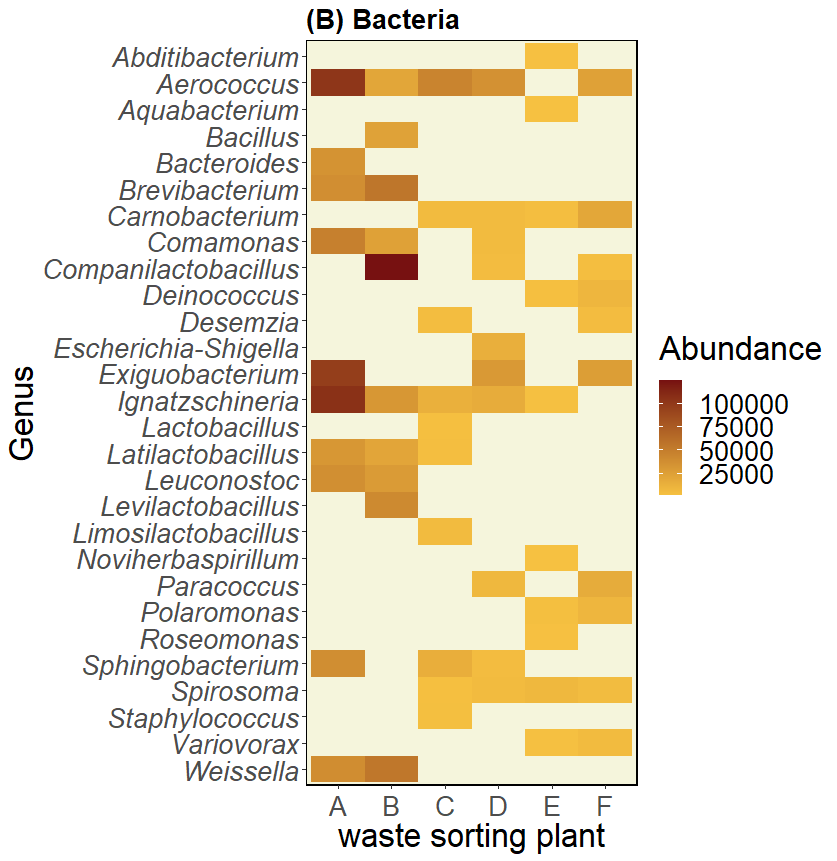


Figure S3 - Heatmap of top 10 abundant fungal (A) and bacterial (B) genera in personal air samples.


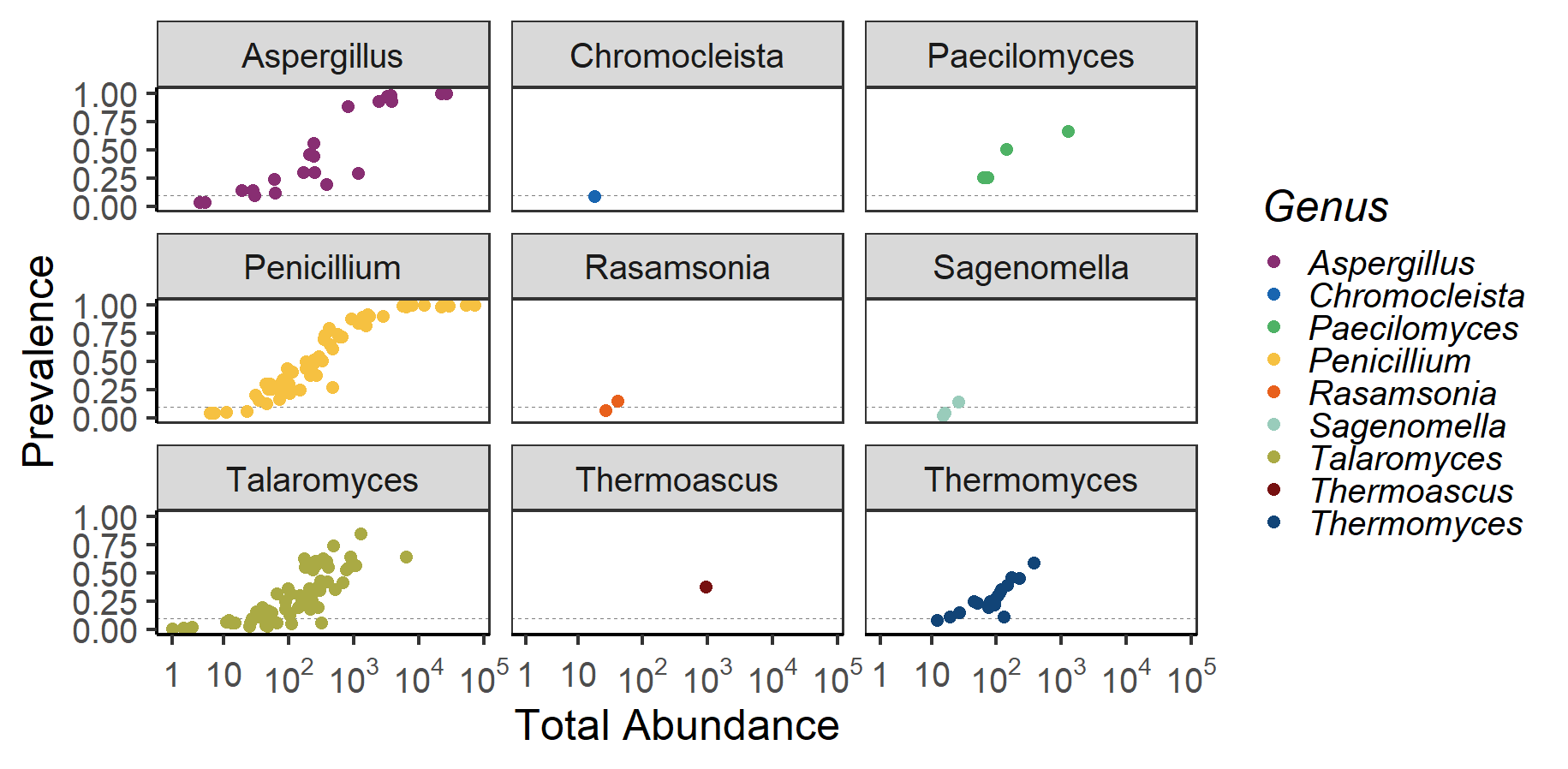


Figure S4 - Prevalence abundance plot of Genera in the order Eurotiales.


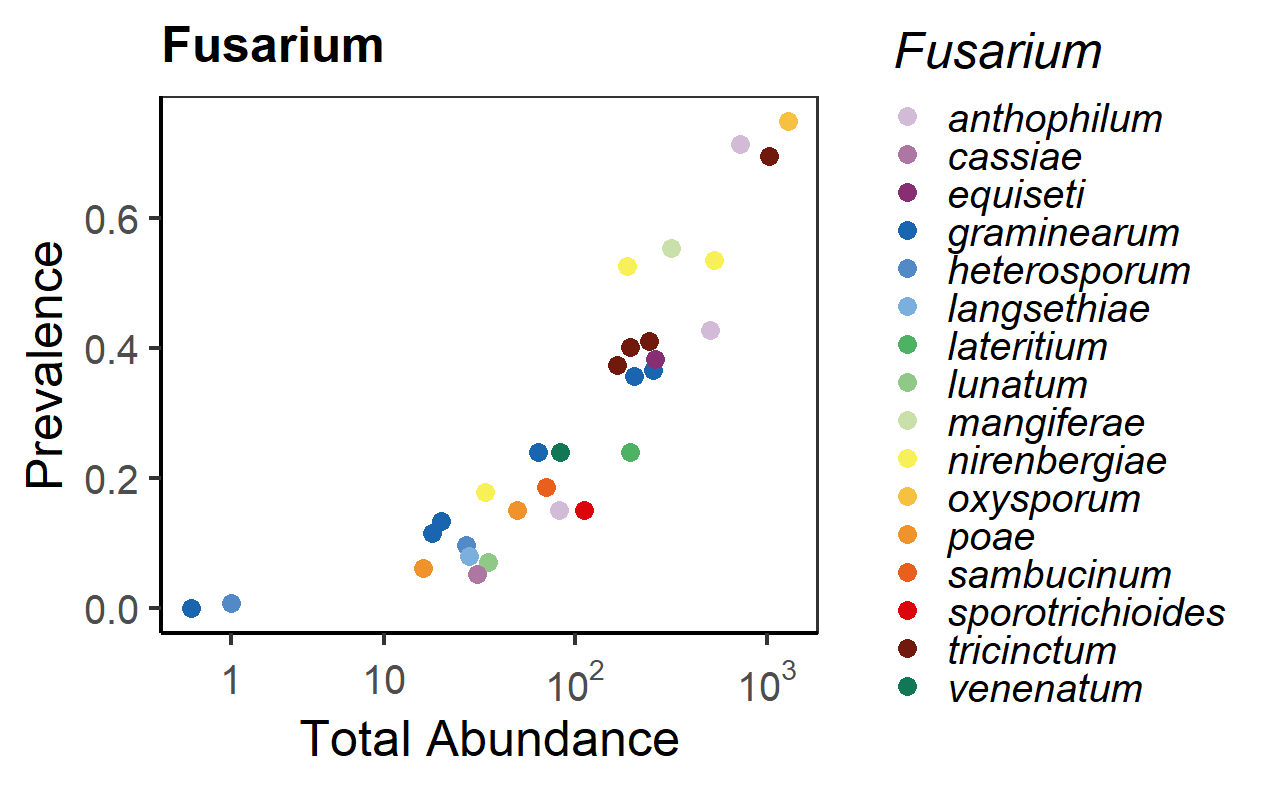


Figure S5 – Prevalence/abundance of species in the genus Fusarium in personal air samples.


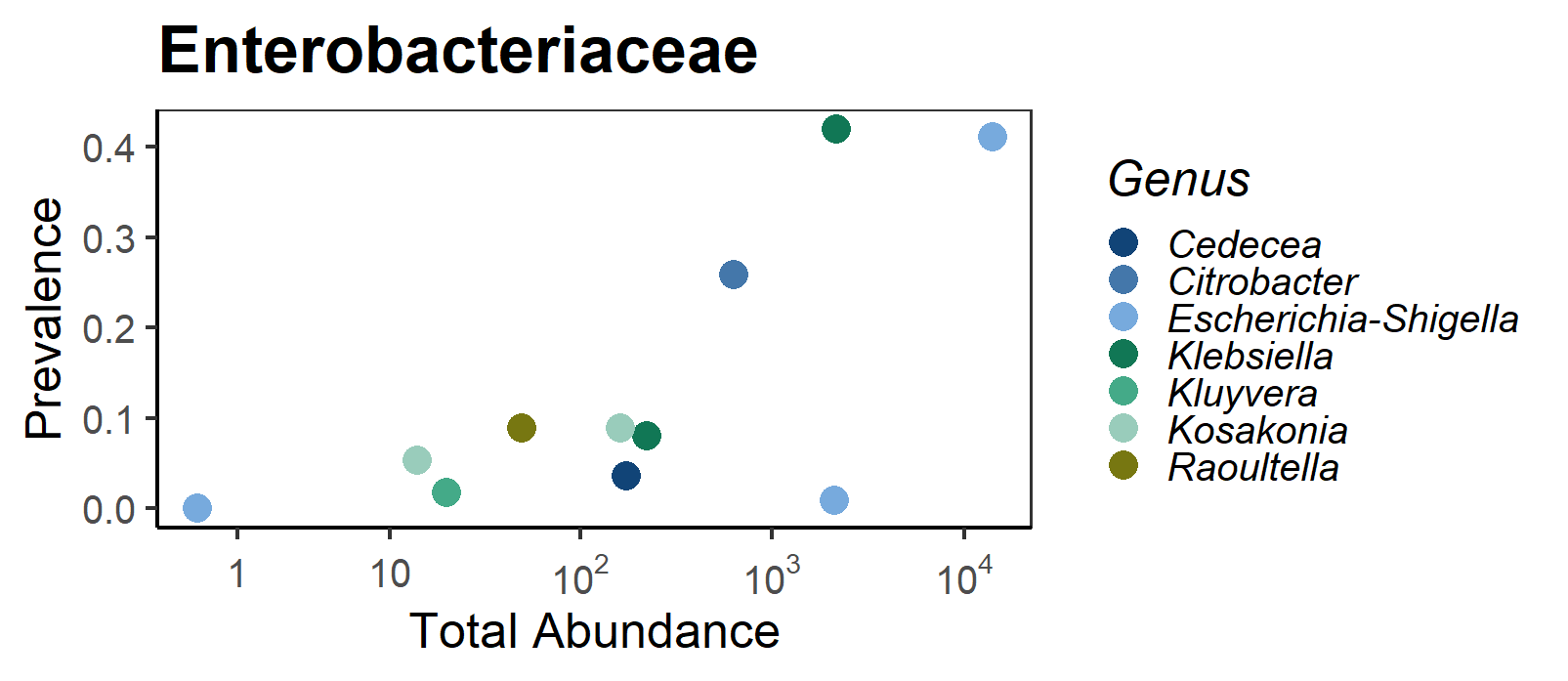


Figure S6 - Prevalence/abundance plot of Enterobacteriaceae in personal air samples.


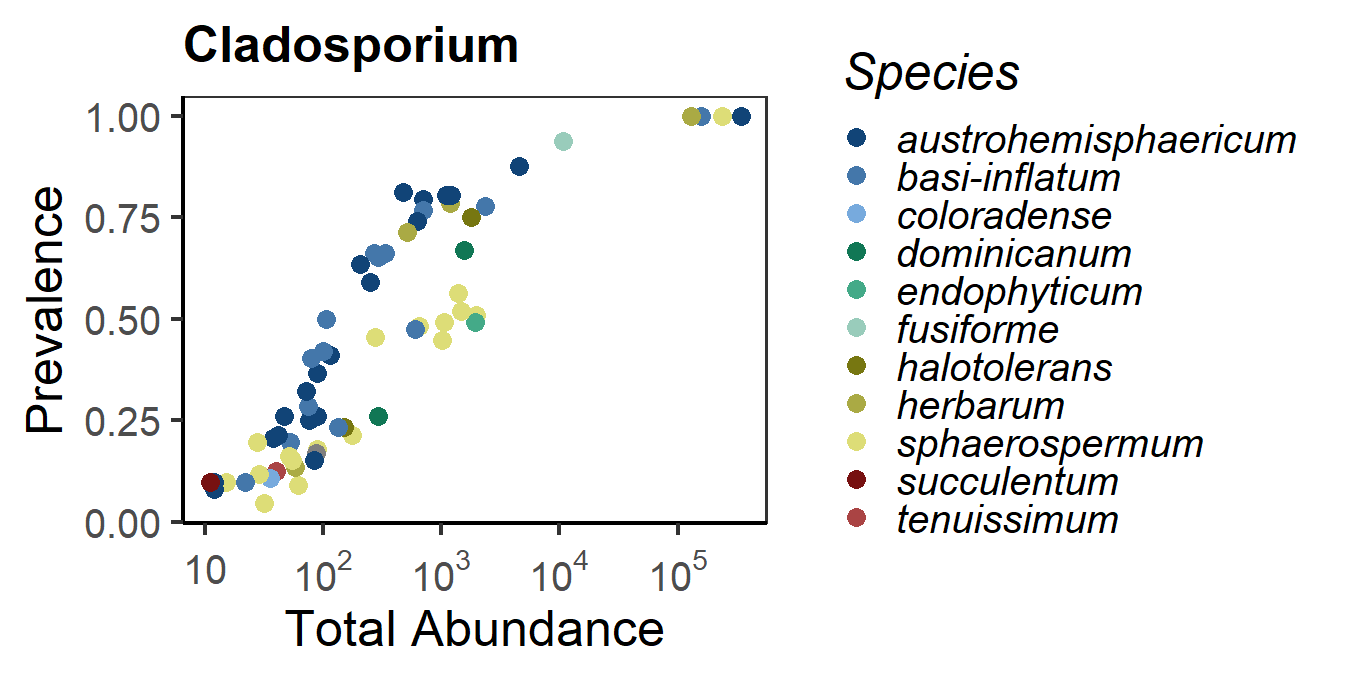


Figure S7 - Abundance/prevalence of Cladosporium in personal air samples.

Table S1- Overview over primers used during PCR and NGS.

| **Target organism** | **Marker** | **Primer** | **Sequence (5’-3’)** | **Reference** |
| --- | --- | --- | --- | --- |
| Universal | 16S rRNA V4-V5 | 515FB | GTGYCAGCMGCCGCGGTAA | (Parada et al., 2016; Walters et al., 2016) |
| universal | 16S rRNA V4-V5 | 926R | CCGYCAATTYMTTTRAGTTT | (Parada et al., 2016; Walters et al., 2016) |
| Fungi-specific | ITS2 | ITS86(F) | GTGAATCATCGAATCTTTGAA | (Op De Beeck et al., 2014) |
| Fungi-specific | ITS2 | ITS4(R) | TCCTCCGCTTATTGATATGC | (Op De Beeck et al., 2014) |

Table S2 – Average temperature (°C) and precipitation (mm) measured at meteorological sites in close proximity to the respective plants during the sampling campaigns. Data retrieved from: https://seklima.met.no/observations/

| **Season** | **Plant** | **Average temperature (°C)** | **Precipitation (mm)** |
| --- | --- | --- | --- |
| Summer | A | 12 | 0.0 |
| Autumn | A | 8.1 | 0.1 |
| Summer | B | 15 | 5.3 |
| Autumn | B | 11 | 15 |
| Autumn | C | 3.7 | 6.5 |
| Autumn | D | 13 | 2.4 |
| Autumn | E | 2.8 | 4.7 |
| Autumn | F | 1.6 | 0 |
